# AI is Here to Stay: A Scenario‐Based Exploration of Leadership, Followership, and the Impact on Skill Development

**DOI:** 10.1002/yd.70067

**Published:** 2026-08-22

**Authors:** Maike Kugler, Laura S. Aichroth

**Affiliations:** ^1^ Business School Pforzheim University Pforzheim Germany; ^2^ Faculty of Business & Management European University for Innovation and Perspective Backnang Germany

## Abstract

Emerging AI technologies are transforming work and redefining how leading and following unfold when humans collaborate with AI as tool, agent, teammate, and autonomous partner. This article uses an action‐based organizational learning scenario technique to develop four future workplace constellations: (1) AI as collaborative tool, (2) AI as delegated agent, (3) AI as team member, and (4) AI as autonomous partner. For each, it examines implications for reversing the lens and co‐creation leadership and followership, specifying the competence profiles and AI literacy needed for responsible human–AI interaction. Comparing these scenario‐specific requirements with current AI literacy levels reveals substantial gaps in leadership and followership education, motivating a stronger emphasis on critical thinking and prompt literacy in development programs.

## 1 Introduction

Emerging technologies are considered a catalyst for change (Ulfert et al. 2024) and the advancements in Artificial Intelligence (AI) offer potential for companies to become more efficient through automation (Ates et al. 2024). On the other hand, the development is slowed down by questions of ethics and energy consumption (Floridi et al. 2018; Lee et al. 2025). Nevertheless, AI is a current trend that practitioners and academics are grappling with, and for companies to benefit from AI in the long term, implementation strategies are needed (Zhang et al. 2021). The role humans are going to play in the workplace is part of the discourse (McAfee and Brynjolfsson 2016; Acemoglu and Restrepo 2019), including the skills relevant in future workplaces (Margaryan 2023).

At the same time, relationships in organizational settings are shifting and roles such as leadership and followership are becoming more complex (Uhl‐Bien 2021). In the last three decades, followership as part of the leadership process has gained attention (Uhl‐Bien et al. 2014; Ribbat et al., 2024) and a more detailed understanding of relational processes in organizations is emerging, although these processes still require further exploration (Riggio 2020). With AI as a new actor in the workplace, the question arises of how leading and following will change.

The interplay between humans and AI can be differentiated into Human‐AI partnership (Ramchurn et al. 2021), more general Human‐AI Collaboration (Khac and Leyer 2026), AI Agents (Sapkota et al. 2026), Human‐AI Teaming (Zhou et al. 2026), and Human‐Autonomy Teaming (Lyons et al. 2021; O'Neill et al., 2022). The question of whether humans and machines are going to lead together or follow each other is crucial in shaping how we live and work in the future (Lai and Rau 2026). Subsequently, the competencies needed for the interaction in future workplaces are impacted by the form of leading and following. Thereby, educators are challenged to review their curricula and include competencies vital for future collaboration (Biagini 2025).

**FIGURE 1:**
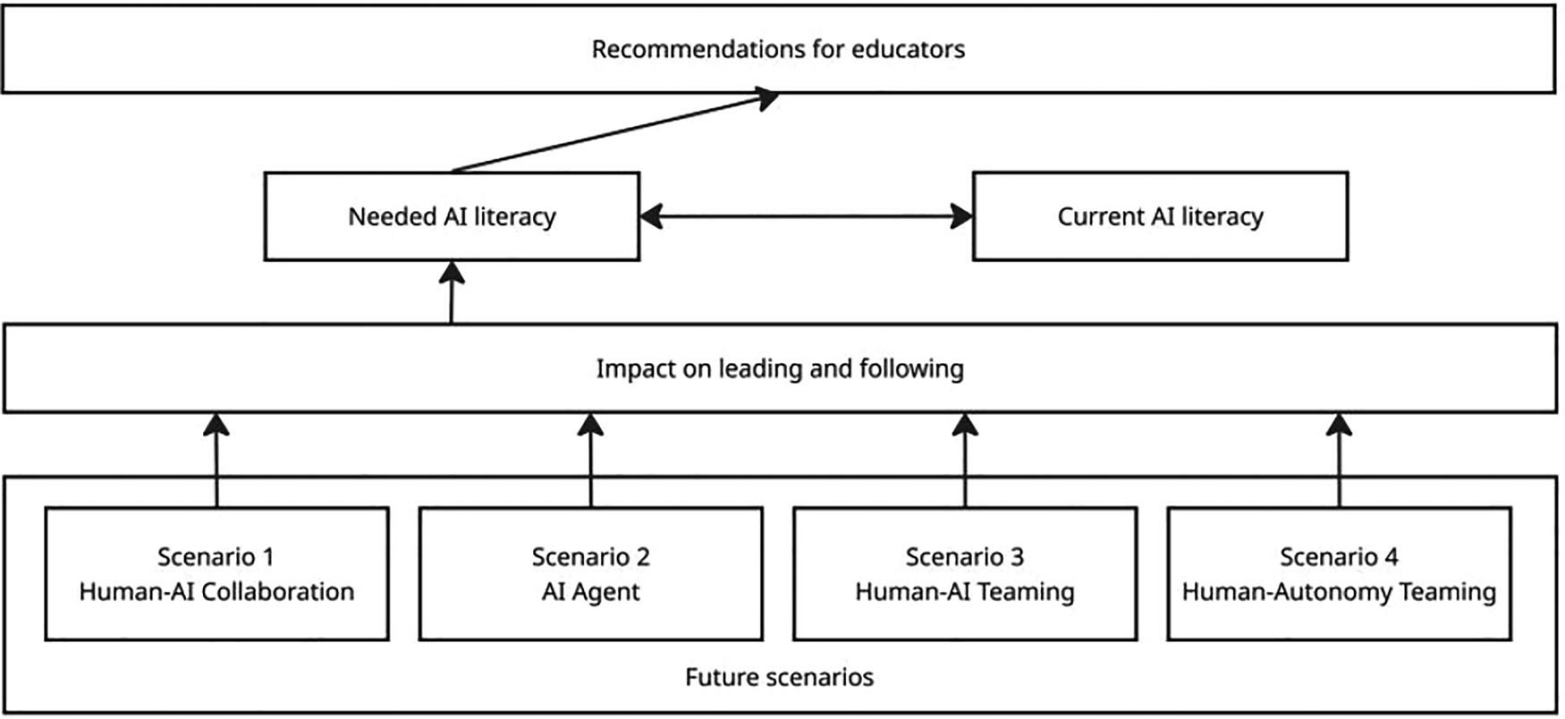
A scenario‐based exploration of leadership, followership, and the impact on skill development. *Note*. Own representation.

Therefore, this article aims to explore the impact AI may have on leading and following in organizations and derive implications for leadership and followership competencies that should be embedded into educational curricula. To address this aim, we take four steps, visualized in Figure 1. First, we derive four potential scenarios from current literature, implementing action‐based organizational learning scenario technique (van der Heijden 2004; Rowland and Spaniol 2022; Wright et al. 2013): (1) AI as a collaborative tool, (2) AI as delegated agent, (3) AI as team member, and (4) AI as autonomous partner. Next, we engage in scenario analysis and reflect on the potential outcomes for leading and following in future organizations. Based on the combination of AI development and its impact on leading and following, we derive a need for AI literacy for each scenario. Finally, we highlight competencies of AI literacy that need to be integrated in educational curricula. Thereby, we provide educators with future‐oriented suggestions for course designs.

## 2 Future AI Development in Organizations

We engaged in scenario thinking using action‐based organizational learning scenario technique, which is the process of envisioning multiple possible futures, each offering different options and necessities for action and leading to diverse outcomes (van der Heijden 2004; Rowland and Spaniol 2022; Wright et al. 2013). Current literature highlights four potential scenarios for the development of AI in the workplace:

### 2.1 Scenario 1: AI as a Collaborative Tool

In the first scenario, which is based on literature on Human‐AI Collaboration (Khac and Leyer 2026), AI implementation remains limited in proactive use and merely assists employees on demand without changing how work is fundamentally performed. Pan et al. (2025) referred to this type of collaboration as Human‐Generative AI Team Collaboration. Although this paper also includes collaboration with generative AI, the shorter name Human‐AI Collaboration is used (Khac and Leyer 2026). Employees are provided with digital services helping them to schedule meetings, draft e‐mails, or prioritize tasks, making daily work more organized and slightly more efficient.

### 2.2 Scenario 2: AI as Delegated Agent

In the second scenario, based on AI Agent literature (Sapkota et al. 2025), also called intelligent agent, software agent, or artificial agent (Kühl et al. 2020), AI is integrated into core workflows and takes over operational tasks. Humans still make key decisions, but AI processes data and identifies efficient ways to implement these decisions, acting as an intelligent executor of human strategic choices. Therefore, Zeiser (2024) calls it AI decision support. While prior literature acknowledges skills as a central factor for the effective development and adoption of AI technologies, there is still limited empirical evidence on the specific skill requirements that arise when AI agents are embedded in workplace practices (Margaryan 2023).

### 2.3 Scenario 3: AI as a Team Member

Human‐AI Teaming (Berretta et al., 2023; Zhou et al. 2026) informs the third scenario. AI becomes a recognized member of the workforce suggesting decisions, task assignment, and feedback. In this emerging mode of work, humans and AI integrate their distinct strengths in a synergistic manner to achieve jointly defined objectives (Berretta et al., 2023). However, the human part of the team is still in charge of final decisions and responsible for goal achievement. Hagemann et al. (2023) emphasize the importance of teamwork characteristics such as being responsive, situational aware, or communicating effectively and point to the further need for research to investigate the processes of enabling human cognition interacting with technical systems.

### 2.4 Scenario 4: AI as Autonomous Partner

The fourth scenario is based on Human‐Autonomy Teaming as an evolution from prior research on human‐automation interaction (Lyons et al. 2021). In contrast to Human‐AI Teaming, Human‐Autonomy Teaming extends beyond advanced automation by granting machines independent decision authority in interdependent team contexts. Humans and autonomous AI systems mutually adapt and coordinate to achieve shared goals (Lyons et al. 2021; O'Neill et al., 2022). Cooperation between humans and autonomous systems is characterised by team‐oriented communication and shared mental models between humans and autonomous systems, which together support the development and maintenance of human‐like team relationships (Lyons et al. 2021).

## 3 Leading and Following in Future Organizations

The leadership process includes two perspectives: leadership and followership (Riggio et al. 2025). While leadership has been explored for more than a decade, followership as part of the process has only been recognized in the last 35 years (Riggio 2020). Leadership and followership can be explored from two general standpoints (Uhl‐Bien et al. 2014, Uhl‐Bien and Carsten 2018, Uhl‐Bien et al. 2026): (1) positional through hierarchy: the reversing the lens perspective, or (2) relational, through social interaction: the co‐creation perspective. From a hierarchical standpoint, leadership is the influence through positional authority (Azad et al. 2017) and followership refers to subordinates without organizational power (Kellerman 2008). From the co‐creation perspective, leadership contains activities and processes of sense‐making leading to relational meaning‐making (Blom and Alvesson 2015) and followership is an interaction between individuals in which they grant each other leader and follower roles (Crossman and Crossman 2011; DeRue and Ashford 2010).

Most of the current literature explores the roles of managers and employees (reversing the lens), while the co‐creation perspective remains underexplored (Uhl‐Bien et al. 2026; Ribbat et al., 2024). For this article, we include both perspectives, acknowledging that leader/manager and employee/follower roles are often adopted by the same organizational members (Blom and Alvesson 2015) but are distinct in their origin and relevant skillset. In the four scenarios, AI‐supported communication is not considered neutral, but rather as being shaped by a technology that can tempt us to relinquish control, raising critical questions regarding responsibility and the capacity to act, which should be considered in each case (Van Quaquebeke and Gerpott 2024). Moreover, to avoid biases or manipulative tendencies and address cultural characteristics, the following scenarios require ongoing consideration of the co‐evolution of humans and AI, understood as an ongoing process in which humans and AI systems influence each other. This co‐evolution is increasingly a hallmark of today's society but has not yet been sufficiently explored in research on artificial intelligence and complexity science (Pedreschi et al. 2025).

### 3.1 Leading and Following in Scenario 1

With AI being a collaborative tool, it serves as a supporting system without its own area of responsibility. Thereby, a new hierarchical level is created in which several AI team members are subordinated to employees. From a reversing the lens perspective, AI adopts follower roles while humans take leadership positions. However, the follower role is situation‐based and does not include autonomous decision‐making or action‐taking. To successfully use AI as a supportive tool, knowledge about prompt structures for effective human‐AI communication is necessary (Federiakin et al. 2024).

From a co‐creation perspective, leadership and followership remain entirely human processes. AI becomes a standard tool present in daily work but does not take part in the social interaction or relationship dynamics that define leadership and followership. However, leadership and followership interaction can be supported and influenced by AI. For example, feedback can be prepared with the help of AI, making it potentially more acceptable (Glickman and Sharot 2025).

It is expected that the development of trust in teams will change as the use of AI for preparing interpersonal communication might influence perceived authenticity (Lee and Mitson 2025). Hence, social and communicative competencies will remain crucial. When such support is used, critical thinking and reflection on AI‐generated content will be relevant before the outcome is used in human interactions (Larson et al., 2024).

### 3.2 Leading and Following in Scenario 2

When AI becomes a delegated agent, it takes a subordinate role while humans are adopting leadership positions. In contrast to Scenario 1, the enactment of followership is strengthened as AI has its own tasks that it performs without situational instruction. Thereby, humans provide the frame conditions through their decisions, and AI operates inside it (Hurwitz and Hurwitz 2015).

To be able to define these frame conditions, knowledge about organizational complexity is necessary. Next, the defined frame conditions need to be transformed into clear prompts, so the AI agent can correctly perform the tasks. Thereby, prompt literacy is needed (Federiakin et al. 2024), extending the knowledge of how to interact with AI. Critical reflection on the output generated by AI is a core competency to effective supervision (Herrmann and Pfeiffer 2023).

From a co‐creation perspective, leading and following remain largely human interactions. Humans might adjust their choices based on the information AI provides. In that limited sense, AI could be interpreted as offering elements of leadership that can be accepted by humans in a relational follower role. For future AI followers, critical reflections are necessary to make AI‐informed choices (Larson et al., 2024).

### 3.3 Leading and Following in Scenario 3

The attributed role of AI changes from that of a tool to that of a team member. Thereby, the relationship between humans and AI is characterized by interdependence rather than one‐sided control. Effective performance in such teams is subject to human and AI coordination and a shared understanding of goals, priorities, and situational constraints.

From a reversing the lens perspective, humans adopt clear leadership positions, while AI follows. Clear assignment of tasks is relevant to coordinate the Human‐AI team (Hagemann et al. 2023). Therefore, leaders need to know the skillsets of their human and articficial subordinates to be able to effectively allocate tasks to the most suitable team member. To do so, they need enhanced knowledge of AI functions and limitations (Federiakin et al. 2024).

In a co‐creation sense, the shift from tool to team member is relevant, as AI can be granted temporary leadership roles. This might be the case when data analysis, pattern recognition or the coordination of complex information is required. In contrast to Scenario 2, this input is not only presented when actively invited but at any point; like any team member can exhibit voice behavior. Again, potential AI followers need to critically review suggestions.

### 3.4 Leading and Following in Scenario 4

Humans and AI operate as equal partners and AI team members have their own area of responsibility. Humans and AI can be appointed positional leader and subordinate roles. With AI in leadership positions, humans remain legally and ethically responsible within today's understanding. Thereby, those who lead AI become an instance of governance control (Lowman 2025). To be able to supervise AI leaders, the human governance instance needs to have a detailed understanding of AI functioning mechanisms (Federiakin et al. 2024).

From a co‐creation perspective, AI can adopt leader and follower roles. These roles and responsibilities are negotiated, and humans and AI influence each other in a shared social and organizational environment. Since followers actively grant leadership (DeRue and Ashford 2010), human acceptance and trust will determine whether AI can take on leadership responsibility in the co‐creation process. Although trust is not a skill, it is connected to critical thinking regarding AI outputs (Kumar et al. 2025).

Interestingly, studies show that people prefer feedback fom AI, but only when they believe it comes from a human (Glickman and Sharot 2025) and AI might be able to address individual needs better than human leaders (van Quaquebeke and Gerpott 2023). Future leaders (human or artificial) will need to develop the ability to adapt their communication style to the team member to foster trust in different ways. While this is already the case for human team members and different styles have been discussed based on follower personality, identity and self (Schyns and Felfe 2006; Van Kippenberg et al. 2004), communication styles towards AI team members might need to be different, less emotional but more precise.

## 4 AI Literacy Gap Analysis

Mills et al. (2024) defined AI literacy as a combination of knowledge and competencies allowing individuals to critically comprehend, assess, and use AI systems and tools in a manner that supports safe, responsible, and effective participation in an increasingly digitalized socio‐technical environment. Almatrafi et al. (2024) derived six constructs relevant for AI literacy: recognize, know and understand, use and apply, evaluate, create, and navigate ethically.

Due to its application‐oriented approach, this paper is based on the definition by Mills et al. (2024) and their threefold approach to “AI literacy includes the knowledge and skills that enable people to critically understand, evaluate, and use AI systems and tools to safely and effectively participate in an increasingly digital world” (p. 4). Based on the analysis of differences in the maturity of AI competencies, MacCallum et al. (2023) suggested four skill levels, shown in Table 1.

**TABLE 1:** AI literacy levels.

AI literacy level	AI literacy category	AI literacy level description
1	Informed	users with introductory awareness and essential, entry‐level understanding
2	Empowered	able to explore ideas and engage critically by reflecting on provided information
3	Engaged	able to co‐design and steer AI‐supported learning by actively implementing and integrating concepts and tools in a collaborative way
4	Active participant	able to design, adapt, and apply AI in ways that drive transformative change

*Note*. Own representation based on MacCallum et al. (2023, p. 4).

These levels capture the spread of digital and AI‐related competencies among students and educators in higher education. Their importance is supported by the findings of Tzirides et al. (2024). They suggest that carefully designed Human‐AI Collaboration in coursework can significantly increase comfort with AI tools, critical understanding of AI's strengths and limitations, and a sense of how to use generative AI responsibly in educational contexts. Tzirides and colleagues highlight remaining gaps and suggest intentional, scenario‐based, and context‐based designs to systematically close the AI literacy gap and prepare students for future jobs.

## 5 AI Future Scenarios and Their Impact on Leading, Following, and AI Literacy

The ongoing development and diffusion of AI in organizational contexts profoundly reshape leading and following and the skills needed to perform them. This transformation poses significant challenges for organizations and their members, as they must develop and adapt both existing and new competencies to succeed in increasingly AI‐mediated work environments (Margaryan 2023). Table 2 shows the integration of the four scenarios with the potential impact on leading and following and the AI literacy levels.

**TABLE 2:** Future AI based scenarios, their impact on leading, following, and necessary competencies and AI literacy.

Scenario	Impact on leading and following	Necessary competencies and AI literacy
AI as collaborative tool	Reversing the lens: Humans in leadership positions, AI in situation‐based followership positions as a tool Co‐creation: Leadership and followership as entirely human processes, supported by AI	Relevant competencies: Understanding prompt structures, some critical reasoning Informed humans
AI as delegated agent	Reversing the lens: Humans in leadership positions, AI in permanent followership positions as a tool Co‐creation: Leadership and followership as largely human interactions, potential for situation‐based AI leadership through suggestions when invited	Relevant competencies: Knowledge of organizational complexity, prompt literacy, critical reasoning, some critical thinking Empowered humans
AI as team member	Reversing the lens: Humans in leadership positions, AI in permanent followership position as a team member Co‐creation: Leadership and followership as human interactions, potential for situation‐based AI leadership through independent suggestions	Relevant competencies: Knowledge of organizational complexity, clear goal definition, prompting literacy, prompting method knowledge, critical reasoning, critical thinking Engaged humans
AI as autonomous partner	Reversing the lens: Humans and AI in leadership and followership positions, humans with legal and ethical responsibility Co‐creation: Leadership and followership as negotiated roles between humans and AI	Relevant competencies: Ethical and legal knowledge, knowledge of organizational complexity, clear goal definition, prompt literacy, prompting method knowledge, critical reasoning, critical thinking Human as active participants

*Note*. Own representation.

It becomes clear how each developmental stage of AI requires more changes in the skill development of humans in the workplace. While Scenario 1 is reality in most organizations today and Scenario 2 is on the rise, not all organization members have been trained to be informed and engaged AI users. The skill gap becomes more apparent when we think about Scenarios 3 and 4, which will change the role humans play in leading and following.

## 6 Recommendations for Educators

AI use is associated with lower levels of critical thinking, an effect that becomes stronger when users place greater trust in AI; however, strong AI literacy substantially weakens this harmful influence of trust (Kulal 2025). These findings indicate that developing AI literacy is a central strategy for adjusting users’ trust to appropriate levels and maintaining their critical thinking regarding AI outputs as a relevant skill. Kulal (2025) therefore highlights the importance of embedding AI literacy programs into education to reduce cognitive vulnerabilities arising from the broad adoption of AI tools. Almatrafi et al. (2024) suggest four aspects of AI empowerment, which are AI self‐efficacy, meaningfulness, impact, and creative self‐efficacy.

Building on this and our scenario analysis summarized in Table 2, educators should derive the specific AI literacy requirements from the most relevant future work and learning scenarios and compare these demands with learners’ current AI literacy levels to identify concrete gaps. On this basis, educational plans and interventions can be systematically adapted to close these gaps, aligning content, methods, and assessments with the competencies needed for safe, critical, and empowering AI use in those future scenarios. In leadership and followership education specifically, this implies designing AI literacy not as a generic digital skill, but as a role‐sensitive competence that enables students to prompt, steer, and codesign AI‐supported processes while being critical thinkers in line with their future responsibilities.

Regardless of the specific scenario, findings by Knoth et al. (2024) indicate more advanced prompt engineering skills are associated with higher‐quality large language models (LLM) outputs, underscoring prompt engineering as an overall key competence for goal‐directed use of generative AI. They also suggest that specific facets of AI literacy support better prompt formulation and the more targeted pedagogical adapion of AI use in educational contexts. Additionally, Federiakin et al. (2024) suggest different levels of prompting knowledge, relevant to varying extents in the scenarios. Based on the analysis, all future organization members should acquire knowledge about prompt structures and develop prompt literacy .

The educational AI literacy framework, also called ED‐AI Lit framework (Allen and Kendeou 2024), adds recommendations for educators as it concepualizes AI literacy as an interdisciplinary educational competence that encompasses understanding, evaluating, and productively using AI in learning environments. They underline the need to embed AI literacy systematically across curricula, emphasizing skills such as critical evaluation, responsible use, and effective collaboration with AI systems rather than treating AI solely as a technical add‐on. Taken together, these insights call for intentional, scenario‐based curriculum design in which AI literacy is embedded across subjects to prepare learners for AI's evolving roles, from a collaborative tool to an autonomous partner, and to support empowered, ethically grounded participation in increasingly AI‐mediated work systems.

Moreover, to align critical pedagogy with social justice aims, it is important to address intercultural dimensions of AI and the ways they can perpetuate or disrupt systemic inequities and the marginalization of certain voices, thereby supporting relational and ethical educational processes that prepare students to become change‐oriented leaders dedicated to fairness, belonging, and well‐being (Yang and Jimenez‐Luque 2025).

## 7 Conclusion

AI's evolving roles, from a collaborative tool to an autonomous partner, will fundamentally reshape leadership and followership in organizations by redistributing agency, redefining hierarchies, and transforming how decisions are made and justified. Across the four scenarios discussed, leaders and followers must increasingly move beyond basic AI use toward steering, scrutinizing, and co‑designing AI‑supported processes as integral parts of their (relational) work. A central strength of this paper is its integrative, future‐oriented perspective that links precise human‐AI work scenarios (from AI as a tool to an autonomous partner) with differentiated AI literacy requirements for leaders and followers. By combining conceptual scenario building with educational implications, this paper offers a structured, practice‐relevant lens for education that goes beyond generic calls for “more AI literacy” and instead specifies different roles needed in distinct future scenarios.

However, although the scenario‐based, action‐oriented design is cognitively insightful and interdisciplinarily derived, it remains an open question how these scenarios can be translated into sustained learning routines or concrete behavioral change. Moreover, the future AI scenarios only represent a limited number of options derived from today's knowledge. Further specification for real‐life, organization‐specific contexts, and future developments will be necessary. To address these limitations, future research should combine scenario work with longitudinal action research to strengthen the translation into behavior and learning routines by systematically tracking how scenario‐derived learning interventions can be piloted or rolled out in organizational or educational practice. To increase contextual precision and real‐life transfer, future research could develop and test sector‐specific “micro‐scenarios” (e.g., for hospitals, universities, or SMEs) and examine how they can be adapted to their structures, contraints, or already existing AI infrastructures or learning routines.

The juxtaposition of the four scenarios with current AI literacy levels provides a scientific contribution by exposing substantial preparedness gaps: many learners potentially remain at introductory stages, while future work contexts require empowered, critically engaged, and ethically grounded AI users. Meeting this challenge calls for deliberate, scenario‑based educational design that derives required competencies from likely human‐AI constellations and aligns learning outcomes, teaching methods, and assessment practices accordingly.

Systematically embedding AI literacy across curricula thus becomes a core task of leadership and followership education, rather than an optional add‑on. Developing such literacy will equip future professionals to calibrate trust, maintain critical thinking, and navigate complex socio‑technical systems, enabling them to harness AI's transformative potential in ways that support responsible, human‐centered, and potentially AI‐team‐member‐centered leadership and followership.
